# Racial and/or Ethnic Differences in Adherence to Preventive Care Guidelines among US Adults Using a Nationally Representative Survey of All Eight Major Racial and/or Ethnic Populations

**DOI:** 10.1007/s40615-026-03159-9

**Published:** 2026-08-21

**Authors:** Paula D. Strassle, Ria A. Warrier, Aidan McGrath Craver, Megan E. Trangsrud, Stephanie A. Ponce, Christian S. Alvarez, Katherine E. Goodman, Cher M. Dallal

**Affiliations:** 1 Department of Epidemiology and Biostatistics, University of Maryland School of Public Health, College Park, MD 20742, USA; 2 Division of Intramural Research, National Institute on Minority Health and Health Disparities, National Institutes of Health, Bethesda, MD, USA; 3 Rollins School of Public Health, Emory University, Atlanta, GA, USA; 4 Department of Epidemiology and Public Health, University of Maryland School of Medicine, Baltimore, MD, USA; 5 University of Maryland Institute for Health Computing, North Bethesda, MD, USA

**Keywords:** Preventive care, Cancer screening, Vaccinations, Health care utilization, 62P25

## Abstract

**Background:**

Preventive care, like cancer screenings, is an effective tool to reduce the risk of morbidity and mortality. Despite these benefits, preventive care is underutilized in the United States and disparities are well-documented. However, preventive care research has largely been limited to Black, Latino, and White populations, and a better understanding of preventive care use across all racial and/or ethnic populations is needed.

**Methods:**

This study used data from the PREVentive Care AdheREnce in Diverse Populations (PREV-CARE) study, a nationally representative, online survey of American Indian or Alaska Native (AIAN), Asian, Black, Latino (English- and Spanish-speaking), Middle Eastern or North African (MENA), Native Hawaiian or Pacific Islander (NHPI), White, and multiracial adults aged ≥35 years (6/2023-8/2023, N=4,111). Preventive care measures included blood pressure screening; cholesterol screening; breast, cervical, and colorectal cancer screening; and influenza, shingles vaccination, and pneumococcal vaccination. Multivariable Poisson regression was used to estimate the association between race and/or ethnicity and adherence to preventive services guidelines. Ethnicity-stratified analyses among Asian, Latino, and NHPI participants were also performed.

**Results:**

Several disparities in vaccination adherence were observed when compared to White adults, among AIAN (influenza PR: 0.82 [95% CI:0.69-0.98]; shingles PR: 0.76, [0.54-1.08]), Black (shingles PR: 0.75, [0.54-1.06]), Spanish-speaking Latino (pneumococcal PR: 0.78 [0.51-1.19]), MENA (influenza PR: 0.75 [0.62-0.92]), and multiracial adults (influenza PR: 0.84 [0.71-1.00]; shingles PR: 0.53 [0.34-0.81]). MENA females were also less likely to undergo cervical cancer screening (PR: 0.85 [0.62-0.92]). Almost all racial and/or ethnic populations were below Healthy People 2030 targets.

**Conclusions:**

Future research should assess how health beliefs and barriers to care contribute to racial and/or ethnic disparities in preventive care.

## Introduction

Preventive care measures, such as cancer screenings, cardiovascular disease screenings, and vaccinations, are effective (and cost effective) ways for individuals to reduce their risk of morbidity and mortality. For example, between 1996 (when breast, cervical, and colorectal cancer screenings were initially recommended by the United States Preventive Services Task Force [USPSTF]) and 2020, breast, cervical, and colorectal cancer screenings have averted 4.75 million cancer deaths [1] and saved $6.5-$8.6 trillion in healthcare spending [2]. Vaccinations have provided similar benefits, with adult vaccinations projected to prevent an estimated 65 million infections and $185 billion in subsequent healthcare costs over the next 30 years [3].

Despite these benefits, preventive care is underutilized in the United States (US) – approximately 1 in 4 adults were not up to date on recommended cancer screenings in 2021 [4] and use of all recommended preventive services has been estimated to be as low as 5.3% in 2020 [5]. The COVID-19 pandemic led to further declines in cancer screening rates [6, 7] and influenza vaccination [8]. Nationally representative estimates of adherence to preventive care guidelines after the official expiration of the COVID-19 Public Health Emergency on May 11, 2023 [9] are still needed to determine whether rates have rebounded to pre-pandemic levels.

Additionally, racial and/or ethnic disparities in adherence to preventive care guidelines are well-documented [8, 10-13], and put certain communities at increased risk of poor health outcomes. These disparities are often due to systemic issues such as access to healthcare, insurance status, access to transportation, and other barriers to care [14-20]. Misinformation may also play an important role [21]. However, most research on adherence has been limited to Black or African American (Black), Hispanic or Latino (Latino), and White populations, with most studies grouping Asian and Pacific Islander populations together and collapsing all other populations into an overly broad “Other” category [22-24]. Studies which provide further disaggregated estimates of preventive care utilization by race and/or ethnicity are needed to better identify gaps in adherence and where culturally appropriate, targeted interventions to increase uptake are needed.

Thus, the objective of this study was to describe race and/or ethnicity specific adherence to preventive care guidelines post-pandemic among a nationally representative sample of US adults that includes Asian, American Indian or Alaska Native (AIAN), Native Hawaiian or Pacific Islander (NHPI), Middle Eastern or North African (MENA), and multiracial adults, as well as Black, Latino and White adults.

## Methods

The PREVentive Care AdheREnce in Diverse Populations (PREV-CARE) survey was administered by YouGov, a consumer research firm in Palo Alto, California. All male and female adults ≥ 35 years old were eligible to participate. YouGov uses a proprietary, opt-in survey panel composed of over 2 million US adults (active) to conduct nationally representative, online surveys. Panel members are recruited via Web advertising, permission-based email campaigns, partner-sponsored solicitations, telephone-to-Web recruitment, mail-to-Web recruitment, and other methods of outreach. Panel members receive incentives through a loyalty program to complete surveys. YouGov, and these methods, have been used previously to conduct nationally representative, online surveys for research in the US [25-27]. The PREV-CARE survey was administered in English and Spanish (Latino sample only); for most questions, previously validated, Spanish translations were available and reviewed by a bilingual research team member. All surveys were completed between June 7, 2023 and August 24, 2023.

To obtain a nationally representative sample within each racial and/or ethnic population, YouGov randomly matched eligible panel members to a target sample using race and/or ethnicity, sex, age, and education. The target sample was drawn from a representative “modeled frame” of US adults, based upon the American Community Survey public use microdata file, public voter file records, the 2020 Current Population Survey Voting and Registration supplements, the 2020 National Election Pool exit poll, and the 2020 Current Employment Statistics surveys. The target sample for the PREV-CARE survey included 500 adults each from AIAN, Asian, Black, Latino, MENA, NHPI, White, and multiracial adults (overall *n* = 4,000). During recruitment, the target quota was increased to 775 for Latino adults to ensure that at least 250 would be Spanish-speaking. The sampling quota for MENA was also unable to be met (final *n* = 336 out of a target 500).

After sample quotas were met for all other racial and/or ethnic populations, survey weights were generated. The matched cases and modeled frame for each racial and/or ethnic population were combined and multivariable logistic regression, adjusting for age, sex, years of education, region, and language preference (Latino only), was used to estimate propensity scores for inclusion in the study. The propensity scores were then grouped into deciles and post-stratified using a three-way stratification of sex, age, education, and language preference (Latino only) to produce the final weight. This combination of matching to a target sample and weighting means that weighted results are national representative within racial and/or ethnic populations (e.g., AIAN participants represent all AIAN males and females 35 years or older living in the US).

### Adherence to Preventive Care Guidelines

Questions from the Medical Expenditure Panel Survey Preventive Care Self-Administered Questionnaire were used to capture information about adherence to preventive care services recommended by the USPSTF (A or B grade) and the Advisory Committee on Immunization Practices. A and B grade preventive care services are the highest levels of recommendation by the USPSTF and are required to be covered without cost-sharing by insurance providers under the Affordable Care Act [20]. For this study, eight preventive services were included: blood pressure screening (performed by a doctor, nurse, or other healthcare professional), cholesterol screening, breast cancer screening (female only), cervical cancer screening (female only), colorectal cancer screening, influenza vaccination, pneumococcal vaccination, and shingles vaccination. Survey participants were only asked questions about preventive services that were recommended for their age group (and sex) based on published guidelines, Supplemental Table 1. Participants also skipped questions if they reported having a contraindication for breast, cervical, or colorectal cancer guidelines; for example, female participants who reported having a hysterectomy or a history of cervical cancer skipped questions about cervical cancer screening. Adherence to colorectal cancer screening was captured using three questions, as multiple screening tools are available (colonoscopy, sigmoidoscopy, fecal occult blood test [FOTB]) and each has a different recommended timeframe. Participants were classified as being adherent if they met the guidelines for at least one colorectal screening tool. The proportion of each racial and/or ethnic population that was eligible for each preventive care guideline (unweighted) and number of contraindications for each cancer screening is reported in Supplemental Table 2.

### Race and/or Ethnicity and Sociodemographics

All participants were asked “Which one of the following would you say best represents your race/ethnicity?” Available responses included Latino/a/x or Hispanic, American Indian or Alaska Native, Asian, Black or African American, Middle Eastern or North African, Pacific Islander, White, and multiracial. Individuals who selected multiracial were then prompted to select all that apply from the above list of racial and/or ethnic populations. AIAN, Asian, Latino, and Pacific Islander participants were also prompted to provide additional information on their ethnicity or national origin. Specifically, Asian participants were asked if they primarily identified as Asian Indian; Chinese; Filipino; Japanese; Korean; Vietnamese; or Other Asian. Latino participants response options included Mexican, Mexican American, Chicano; Puerto Rican; Cuban/Cuban American; Dominican; Central American; South American; and Another Hispanic, Latino, or Spanish origin, and NHPI participants were asked to identify as Guamanian or Chamorro; Native Hawaiian; Samoan; or Other Pacific Islanders. Latino participants were also stratified by their survey language preference into English- and Spanish-speaking Latino; self-reported limited English proficiency was significantly correlated with English- versus Spanish-speaking status (5.3% vs. 44.3%, *p* < 0.0001).

Self-reported age, sex, English language proficiency, highest education, household annual income, access to a private vehicle, urbanicity, Census division, general health, chronic health conditions, height (feet and inches) and weight (pounds) (to calculate body mass index [BMI, kg/m^2^]), and disability status were also collected. Limited English proficiency was classified as being able to speak English “not at all”, “poorly”, or “fairly well”. Missing data was minimal for all questions (< 1%) except for household annual income (348 participants [unweighted] reported “Prefer not to say”).

### Statistical Analyses

Adherence to preventive services guidelines was estimated across race and/or ethnicity (and language for Latino participants) and other sociodemographic characteristics. Race and/or ethnicity (and language) stratified cancer screening and influenza vaccination rates were then compared to 2030 Healthy People targets: breast cancer screening (80.3%) cervical cancer screening (79.2%), colorectal cancer screening (72.8%), and influenza vaccination (70.0%) [5].

Multivariable Poisson regression with robust variance estimates was used to estimate the association between race and/or ethnicity (and language) and adherence to each preventive services guideline after adjusting for age (continuous), sex (excluding breast and cervical cancer screenings), chronic health conditions (any vs. none), and self-reported health (excellent/very good/good vs. fair/poor). Variables related to access to care (e.g., health insurance status) were not included in the models because it was hypothesized these factors would serve as mediators and potentially explain racial and/or ethnic disparities in adherence. For comparison purposes [28], this study also reports associations between race and/or ethnicity and adherence to all recommended guidelines; the same adjustment set was used. White participants were used as the reference population for all analyses because they represent the majority population in the geographic context (United States) and had the highest adherence to all recommended guidelines among the 8 major racial and/or ethnic populations.

As a secondary analysis, ethnicity/national origin stratified estimates of adherence to preventive care guidelines, comparison to Healthy People 2030 targets, and multivariable regression were also performed among Asian, Latino, and NHPI participants. Ethnicity-stratified analyses could not be performed for AIAN due to the low prevalence of Alaska Native (< 5%). Analyses where the eligible population for a specific guideline was < 11 (unweighted) were not reported due to generalizability concerns. Sample sizes for each ethnicity/national origin stratified analysis are reported in Supplemental Table 2.

All analyses were performed using SAS version 9.4 (SAS Inc., Cary, NC). All analyses were weighted to produce nationally representative estimates within each racial and/or ethnic population, and counts were rounded.

## Results

There were 4,287 online survey respondents (response rate: 30.9%) who were matched down to a sample of 4,111 individuals to produce the final, weighted PREV-CARE data set. Demographics of PREV-CARE participants, stratified by race and/or ethnicity, are reported in Table 1. Overall, adherence to preventive services guidelines ranged from 51.0% (shingles vaccine) to 85.5% (blood pressure screening), Fig. 1.

### Adherence to Cardiovascular Disease Screenings

Self-reported adherence to blood pressure screening guidelines was ≥ 80% among all racial and/or ethnic populations (Table 2) and Asian, Latino, and NHPI ethnicity/national origin subpopulations (Supplemental Tables 3-5). Prevalence of blood pressure screening increased with age (e.g., 78.1% among those 35–39 years old compared to 96.1% among those 75+) and income (<$20,000: 79.8% vs. ≥$100,000: 88.8%), Supplemental Table 6. Adherence was also substantially higher among those with health insurance (any private insurance: 88.6%; public insurance only: 88.4%), compared to uninsured adults (61.7%), and among those with chronic health conditions (91.7% vs. 75.8%) and disabilities (90.1% vs. 83.3%). After adjusting for age, sex, chronic conditions, and self-reported health, no significant differences in blood pressure screening were observed, Table 3.

Self-reported adherence to cholesterol screening was also ≥ 80% for all racial and/or ethnic populations besides AIAN adults (77.3%, Table 2) and Asian, Latino, and NHPI ethnicity/national origin subpopulations (Supplemental Tables 3-5). After adjustment, no significant differences were observed across race and/or ethnicity, Table 3. Similar to blood pressure screening, the prevalence of cholesterol screening also increased with age (72.8% among those 35–39 year olds to 96.3% among those 70–74); a smaller increase was also observed across income (<$20,000: 80.9% vs. ≥$100,000: 85.0%), Supplemental Table 6. Adults with health insurance were also more likely to undergo cholesterol screening (any private: 86.0%; public insurance only: 85.0%; uninsured: 62.3%).

### Adherence to Cancer Screenings

Adherence to breast, cervical, and colorectal cancer screenings were consistently the lowest among MENA populations (breast: 53.4%; cervical: 55.7%; colorectal: 64.8%), Table 2. Less consistency was observed across Asian, Latino, and NHPI ethnicity (Supplemental Tables 3-5). For example, breast cancer screening was lowest among Japanese females (53.2% vs. 70.0–90.6%), whereas cervical cancer screening was lowest among Chinese (54.4%) and Korean (55.1%) females (other ethnicities ranged from 61.8 to 79.7%). Both breast cancer and colorectal cancer screening adherence increased with age, Supplemental Table 6. Adults with chronic conditions, those with public or private health insurance, and those with higher annual household incomes were also more likely to be adherent to cancer screening guidelines.

Only Spanish-speaking Latina females met the 2030 Healthy People breast cancer screening target, and no racial and/or ethnic populations met the target for cervical cancer screening, Table 4. Asian, Black, Spanish-speaking Latino, and White adults met the 2030 Healthy People target for colorectal cancer screening. When results were further stratified by ethnicity, Asian Indian, Chinese, Filipino, Puerto Rican, Guamanian or Chamorro, and other Pacific Islander participants met breast cancer screening guidelines; Puerto Rican, South American and Other Latino participants met cervical cancer screening guidelines; and Asian Indian, Chinese, Filipino, Cuban, and Puerto Rican participants met colorectal cancer screening guidelines, Supplemental Table 7.

After adjustment, Spanish-speaking Latina females still appeared marginally more likely to undergo breast cancer screening (PR 1.26, 95% CI 0.93–1.70), and Black females more likely to undergo both breast cancer (PR 1.10, 95% CI 0.84–1.45) and cervical cancer (PR 1.17, 95% CI 0.89–1.54) screening, compared to White females, although the confidence intervals were wide, Supplemental Table 3.

### Adherence to Vaccinations

Influenza, pneumococcal, and shingles vaccinations were consistently the lowest reported preventive care by all racial and/or ethnic populations, Table 2. Less than half of AIAN (46.5%), MENA (41.4%), and multiracial (45.8%) adults reported getting the influenza vaccine in the past year, and no racial and/or ethnic population met the 2030 Healthy People target for influenza vaccination, Supplemental Table 5. When stratified by ethnicity, only Filipino participants met the 2030 Healthy People target, Supplemental Table 7. Low adherence to shingles vaccine guidelines was also observed in several populations (AIAN: 42.6%, Black: 43.6%, multiracial: 29.6%). Adherence to pneumococcal vaccination guidelines, which had the highest adherence of the vaccines (overall: 69.2%), was lowest among AIAN (56.3%) and Spanish-speaking Latino (52.9%) adults.

After adjustment, AIAN (PR 0.82, 95% CI 0.69–0.98), MENA (PR 0.75, 95% CI 0.62–0.92), and multiracial (PR 0.84, 95% CI 0.71–1.00) adults were still significantly less likely to receive the influenza vaccine compared to White adults, Table 3. After adjustment, lower vaccination rates also persisted among Black (PR 0.75, 95% CI 0.54–1.06) and multiracial (PR 0.53, 95% CI 0.34–0.81) adults for the shingles vaccine compared to White adults.

When looking at other sociodemographic characteristics, influenza vaccination increased with age (42.2% among adults 35–39 years old increased to 80.7% among adults 75+) and family annual income (44.5% among those making <$20,000 up to 57.3% among those making ≥$100,000), Supplemental Table 2. Influenza vaccination was also higher among those with chronic conditions (58.9% vs. 42.1%), insurance (any private: 55.7%, public insurance only: 54.8%, uninsured: 28.4%), not limited English proficiency (52.9% vs. 44.1%), and who graduated from college or attended graduate school (56.7% and 65.2%, respectively, compared to 47.3–51.0% some college/vocational school or less than high school graduate). Pneumococcal and shingles vaccinations were also higher with increasing age, those with chronic conditions, those with insurance, higher education, and higher family annual income. Pneumococcal and shingles vaccinations were also higher among adults living in New England (pneumococcal: 89.4%; shingles: 72.5%), compared to other Census divisions (pneumococcal range: 51.7–74.1%; shingles range: 37.0–58.6%).

### Adherence to All Preventive Care Guidelines

Overall, only 34.4% of survey participants were adherent to all their recommended preventive care guidelines and disparities were observed across race and/or ethnicity after adjustment, with AIAN (PR 0.69, 95% CI 0.56–0.86), MENA (PR 0.64, 95% CI 0.50–0.82), and multiracial (PR 0.79, 95% CI 0.64–0.97) participants being significantly less likely to be adherent to all recommended guidelines, compared to White participants. Adherence to all preventive care guidelines by race and/or ethnicity are reported in Supplemental Table 6.

## Discussion

In a nationally representative, online survey conducted in summer 2023 that included disaggregated estimates for adults from all eight major US racial and/or ethnic populations (as defined by the US Office of Budget and Management [OMB]), this analysis found notable differences in adherence across race and/or ethnicity, as well as other sociodemographic characteristics. AIAN, Black, MENA, and multiracial adults were less likely to be adherent to at least one vaccination guideline compared to White adults. Conversely, Black and Spanish-speaking Latina females were more likely to undergo breast and cervical (Black females only) cancer screenings. Despite these differences, most racial and/or ethnic populations (and subpopulations) were below Healthy People 2030 targets for cancer screening and influenza vaccination. Having insurance, being older, having higher annual household income, having chronic conditions, and having disabilities were also consistently associated with adherence to preventive care guidelines. To the best of our knowledge, this is one of the most recent assessments on the prevalence of adherence to preventive care in the United States, and the first to include AIAN, MENA, NHPI, and multiracial adults – populations that are typically aggregated with other populations (or included as ‘other race’) in previous preventive care research – and report ethnicity/national origin stratified results for Asian, Latino, and NHPI adults.

Overall, our national estimates of adherence to preventive care guidelines were largely similar to recently reported estimates. For example, Star et al. found a similar estimate for adherence to breast cancer screening (*n* = 6,829) in their cross-sectional study of the 2023 National Health Interview Survey (NHIS) (64.9% vs. 69.8% in this study) [29]. This study also found similar adherence for the pneumococcal (64.0% vs. 69.2% in this study), shingles (48.0% vs. 51.0% in this study), and influenza (57.8% vs. 52.3% in this study) vaccinations compared to an analysis of the 2022 NHIS and 2022–2023 influenza season [22]. Estimates for blood pressure (84.0% [24] vs. 85.5%) were also similar to estimates obtained using 2021 NHIS data [24]. However, many recent assessments of preventive care have assessed screening in the past year rather than adherence to preventive care guidelines, which gives an incomplete picture of preventive care use, especially for screenings that only need to be performed every five or ten years. For example, screening within the past year for cervical (40.9% vs. 69.1% in this study) and colorectal (24.3% vs. 71.1% in this study) cancer and cholesterol screening (68.8% vs. 82.8% in this study) are much lower than our adherence estimates [24]. Our ability to assess recent/current adherence to preventive care guidelines, rather than screening within the past year, fills a critical gap in our current understanding of preventive care utilization.

The findings from this study and others [22, 24, 29] highlight that there are significant areas of improvement in preventive care utilization in the United States. And while all racial and/or ethnic groups would benefit from increased cancer screening and vaccination, AIAN, MENA, and multiracial populations would likely see an outsized effect on health outcomes given documented disparities. Studies have shown that culturally tailored interventions [30, 31] and health communication [32] can increase healthcare utilization within racial and/or ethnic populations and other communities. However, additional research is needed to understand how to reduce barriers and facilitate action [32, 33], and future research should focus on addressing these gaps in order to improve preventive care utilization and reduce health disparities.

Various individual and systemic barriers to care likely contribute to suboptimal adherence to preventive care guidelines in the US, but there is limited evidence on the effects of specific barriers on preventive care utilization or the effectiveness of interventions [22]. To date, most studies have focused on health insurance coverage and cost as the primary cause for nonadherence, especially for cancer screenings [23]. However, since the 2010 Affordable Care Act was passed, which expanded health insurance coverage and required insurance providers to cover preventive services free of charge, only modest increases in cancer screening rates have been observed [24, 25] – indicating that insurance status/cost is not the only barrier individuals experience.

Perceived benefits (or lack thereof) also likely contribute to suboptimal adherence, especially for vaccination rates. Previous studies have suggested that the dramatic increase in misinformation and distrust (particularly around vaccinations) on social media during the COVID-19 pandemic has also spilled over into other vaccine-preventable diseases, including influenza [26, 27]. Ability to take time off work [16], housing instability [34], food insecurity [18, 34, 35], transportation barriers [16, 34, 35], socioeconomic status [16, 18, 20, 35], and incarceration history [36] have also been linked to lower adherence to cancer screenings and vaccinations. Additional research is needed to understand the multifactorial-multilevel causes of suboptimal adherence to preventive care guidelines across racial and/or ethnic populations, in order to identify modifiable risk factors that can be targeted for intervention and identify high-risk populations that may benefit most from culturally tailored programming.

Both systemic barriers to care [14] and health misinformation [21] have been found to create or exacerbate racial and/or ethnic disparities in healthcare utilization, and may explain the disparities in adherence to preventive care observed in our study and others [14, 16]. Notably, the largest disparities in adherence observed in this study were among AIAN and MENA populations. Future studies should take steps to investigate these communities in healthcare utilization and outcomes research to better understand their specific needs and barriers. For example, while AIAN populations are more likely to be insured than White adults because of the Indian Health Service (IHS), geographical isolation, limited access to IHS facilities, and other systemic issues may explain lower healthcare utilization and preventive care use in AIAN populations [37]. Previous studies also suggest that perceived negative attitudes toward Muslims has been linked to poorer access to healthcare, and MENA individuals have reported higher rates of perceived healthcare discrimination when seeking healthcare [38]. A deeper understanding (and targeted solutions) of the barriers specific communities, like AIAN and MENA, face could lead to the largest improvements in healthcare utilization, including preventive care, among high-risk populations.

### Strengths and Limitations

This study has several notable strengths. First, the PREV-CARE survey was conducted to generate nationally representative estimates of adherence to preventive care guidelines within the eight major racial and/or ethnic populations in the U.S., as defined by the updated 2024 OMB standards. The PREV-CARE sampling quotas (initially *n* = 500 for each racial and/or ethnic population) allowed us to recruit enough AIAN, NHPI, and MENA individuals to estimate adherence to preventive care guidelines within these populations, as well as stratify Latino adults by English- and Spanish-speaking status. For comparison, the Medical Expenditure Panel Survey (MEPS) and National Health Interview Survey (NHIS) include < 500 AIAN adults, NHPI is combined with Asian, and MENA adults are coded as White in currently available data. This study also used previously validated survey measures to capture adherence to preventive care.

This study also has some limitations. First, this survey was administered online, and individuals with limited digital literacy and/or internet access may have been less likely to participate, potentially introducing selection bias. Lower digital literacy has been associated with many health outcomes, including higher prevalence of chronic diseases and poorer long-term disease management [39], and it is possible that those with low digital literacy may also be less likely to adhere to preventive care guidelines. And while participants were matched and weighted on sociodemographics to obtain nationally representative estimates, some selection bias may exist, and our results may overestimate adherence to preventive care in the U.S. The ethnicity-stratified results among Asian, Latino, and NHPI participants may be particularly prone to bias, due to small sample sizes; this survey was also not designed to be national representative within these groups. Second, the survey was administered in English and Spanish (Latino participants only), and non-Latino participants with low English proficiency were also less likely to participate. Low English proficiency is a known barrier to healthcare, and studies have found that non-English speaking adults are less likely to adhere to preventive care guidelines [40]. This may especially impact our findings for Asian adults; in our study only 9.2% self-reported limited English compared to 26.2% in recent national estimates [41]. Ethnicity-stratified analyses among AIAN and multiracial adults were also not performed. Future studies should assess differences in adherence to preventive care guidelines and potential barriers to care among these populations. We also acknowledge the limitations of using White participants as reference; [42] however, Healthy People 2030 targets were not available for all preventive care guidelines included in this analysis, and had to defer to comparisons across racial and/or ethnic groups. Finally, adherence to preventive care was self-reported and may be subject to nondifferential misclassification.

### Public Health Implications

While adherence to some recommended preventive care guidelines appears to be increasing, adherence to cancer screenings remains below the Healthy People 2030 target in almost every racial and/or ethnic population and all racial and/or ethnic populations are below the 2030 Healthy People influenza vaccination target. Substantial disparities in adherence to preventive care also exist, particularly among AIAN, MENA, NHPI, and multiracial adults. Our nuanced assessment of preventive care across all eight major racial and/or ethnic populations provides critical information for identifying high-risk populations and reducing the impact of disease in the U.S., especially among AIAN, MENA, and NHPI populations. Future research should assess how health beliefs and systemic barriers to care influence adherence to preventive care guidelines across race and/or ethnicity and other sociodemographic characteristics so culturally tailored interventions and health communication strategies can be developed. In the interim, there should be a heightened focus on ensuring all individuals have access to preventive care. Physicians and healthcare providers should recognize cultural differences in preventive care access, health behaviors, and health beliefs to better support patients as they seek preventive care and provide information to assuage any concerns.

## Supplementary Material

Supplement

**Supplementary Information** The online version contains supplementary material available at https://doi.org/10.1007/s40615-026-03159-9.

## Figures and Tables

**Fig. 1:**
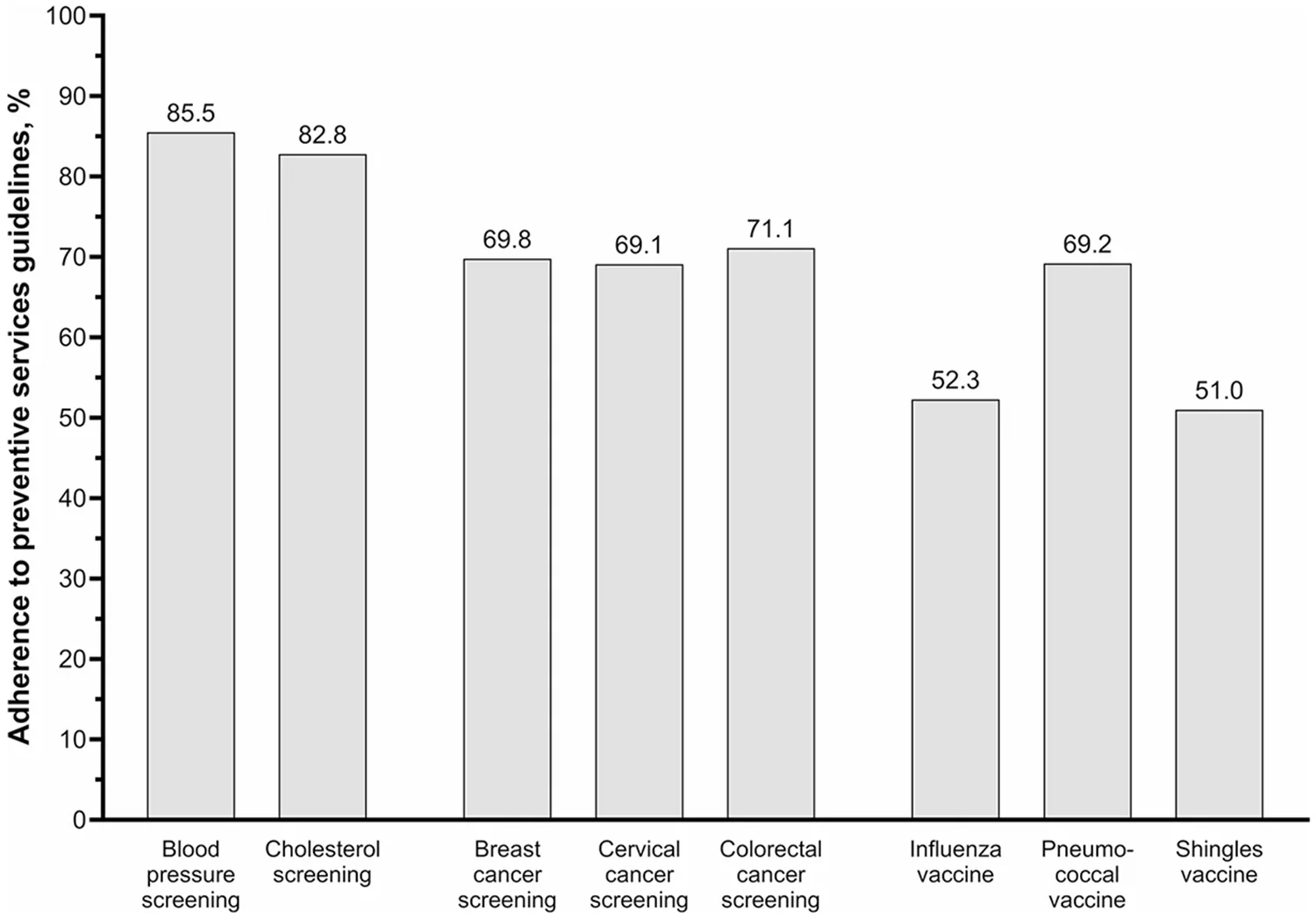
Adherence to preventive services guidelines, weighted to be nationally representative within racial and/or ethnic populations: United States, June – August 2023 (N=4,111)

**Table 1:** Sociodemographic and health-related characteristics of PREV-CARE survey participants, stratified by race and/or ethnicity, weighted to be nationally representative within racial and/or ethnic populations: United States, June – August 2023 (*N* = 4,111)

	Latino								
	AIAN	Asian	Black	English-speaking	Spanish-speaking	MENA	NHPI	White	Multiracial
Total, N	500	500	500	402	373	336	500	500	500
Age, years, median (IQR)	53 (43, 64)	52 (42, 63)	54 (43, 63)	52 (43, 62)	49 (42, 58)	52 (43, 63)	53 (42, 63)	59 (47, 69)	49 (41, 60)
Male, n (%)	242 (48.3)	226 (45.2)	221 (44.2)	220 (54.8)	158 (42.3)	180 (53.5)	246 (49.3)	239 (47.8)	237 (47.5)
Self-reported general health, n (%)									
Excellent\very good\good	339 (67.7)	276 (55.2)	329 (65.7)	250 (62.2)	187 (50.2)	174 (51.7)	327 (65.5)	305 (61.1)	315 (63.0)
Poor\fair	161 (32.3)	224 (44.8)	171 (34.3)	152 (37.8)	186 (49.8)	162 (48.3)	173 (34.5)	195 (38.9)	185 (37.0)
Chronic condition(s)^a^, n (%)	347 (69.4)	227 (45.4)	322 (64.5)	231 (57.5)	200 (53.7)	196 (58.4)	327 (65.3)	331 (66.2)	321 (64.1)
BMI^b^, n (%)									
Underweight (< 18.5)	3 (0.6)	14 (2.7)	5 (1.0)	2 (0.4)	8 (2.1)	4 (1.2)	7 (1.5)	11 (2.3)	4 (0.8)
Normal weight (18.5–24.9)	117 (23.4)	264 (53.0)	124 (24.8)	88 (22.1)	87 (23.3)	95 (28.5)	125 (25.0)	125 (25.3)	136 (27.6)
Overweight (25.0–29.9)	173 (34.6)	148 (29.7)	157 (31.4)	145 (36.2)	137 (36.7)	131 (39.2)	149 (30.0)	171 (34.4)	166 (33.5)
Obese (30.0–39.9)	151 (30.2)	63 (12.6)	156 (31.2)	142 (35.6)	123 (33.0)	84 (25.1)	155 (31.1)	150 (30.1)	141 (28.4)
Severe obesity (40.0+)	55 (11.1)	10 (1.9)	58 (11.6)	23 (5.7)	18 (4.9)	20 (6.0)	62 (12.4)	40 (8.0)	48 (9.7)
Any disability^c^, n (%)	210 (42.0)	104 (20.9)	149 (29.7)	102 (25.5)	78 (20.8)	111 (33.0)	171 (34.2)	177 (35.4)	187 (37.4)
Health insurance, n (%)									
Any private	175 (35.2)	324 (64.9)	212 (42.4)	180 (44.8)	131 (35.6)	173 (51.8)	230 (46.2)	259 (52.2)	228 (45.5)
Public insurance only	269 (53.9)	149 (29.9)	226 (45.1)	171 (42.5)	138 (37.7)	112 (33.4)	224 (45.0)	212 (42.8)	224 (44.7)
Uninsured	54 (10.9)	26 (5.3)	63 (12.5)	51 (12.6)	98 (26.7)	50 (14.8)	44 (8.9)	25 (5.0)	49 (9.8)
Limited English proficiency^d^, n (%)	11 (2.2)	46 (9.2)	7 (1.3)	21 (5.3)	165 (44.3)	21 (6.1)	15 (3.0)	3 (0.5)	8 (1.5)
Education, n (%)									
Less than high school graduate	52 (10.3)	10 (2.1)	31 (6.2)	29 (7.2)	42 (11.3)	21 (6.3)	33 (6.7)	11 (2.1)	33 (6.7)
High school diploma/GED	174 (34.7)	116 (23.2)	181 (36.1)	203 (50.6)	132 (35.3)	77 (23.1)	170 (34.1)	151 (30.1)	122 (24.3)
Some college/vocational school	174 (34.7)	89 (17.9)	153 (30.6)	114 (28.4)	98 (26.1)	63 (18.7)	159 (31.8)	133 (26.5)	166 (33.2)
College graduate	65 (13.1)	158 (31.6)	82 (16.5)	35 (8.7)	71 (18.9)	99 (29.5)	93 (18.7)	121 (24.2)	107 (21.4)
Graduate or advanced degree	36 (7.2)	126 (25.2)	53 (10.6)	21 (5.2)	31 (8.4)	75 (22.5)	44 (8.8)	85 (17.0)	73 (14.5)
Employment status, n (%)									
Employed (full or part-time)	211 (42.2)	301 (60.2)	219 (43.8)	190 (47.2)	210 (56.7)	185 (55.0)	255 (51.0)	210 (41.9)	249 (49.8)
Retired	121 (24.2)	105 (21.0)	127 (25.3)	95 (23.5)	43 (11.5)	70 (20.7)	101 (20.2)	177 (35.5)	84 (16.8)
Not employed^e^	289 (57.8)	199 (39.8)	281 (56.2)	212 (52.8)	160 (43.3)	151 (45.0)	245 (49.0)	290 (58.1)	251 (50.2)
Marital status, n (%)									
Married/domestic partnership	202 (40.4)	307 (61.5)	189 (37.9)	221 (54.8)	226 (60.5)	206 (61.2)	244 (48.7)	281 (56.2)	235 (47.0)
Separated/divorced/widowed	170 (34.1)	77 (15.4)	120 (24.1)	88 (22.0)	90 (24.0)	64 (19.2)	148 (29.6)	112 (22.5)	108 (21.6)
Never married	128 (25.5)	116 (23.1)	190 (38.0)	93 (23.2)	58 (15.4)	66 (19.6)	109 (21.7)	107 (21.3)	157 (31.5)
Family annual income, n (%)									
<$20,000	133 (18.4)	53 (7.3)	103 (14.1)	62 (8.6)	107 (14.8)	42 (5.7)	83 (11.4)	59 (8.2)	84 (11.6)
$20,000-$59,999	203 (13.5)	124 (8.2)	194 (12.8)	171 (11.3)	150 (10.0)	137 (9.1)	185 (12.3)	170 (11.3)	175 (11.6)
$60,000-$99,999	80 (9.7)	119 (14.4)	87 (10.5)	84 (10.2)	74 (9.0)	59 (7.1)	110 (13.3)	115 (13.9)	99 (12.0)
≥$100,000	51 (7.2)	150 (20.9)	71 (10.0)	57 (8.0)	29 (4.1)	81 (11.3)	77 (10.8)	107 (14.9)	92 (12.8)
Access to private vehicle, n (%)									
All of the time	365 (73.1)	398 (79.5)	347 (69.3)	310 (77.1)	240 (64.3)	274 (81.6)	384 (76.8)	448 (89.8)	373 (74.5)
Some of the time	84 (16.9)	70 (14.0)	85 (17.0)	57 (14.3)	79 (21.2)	37 (10.9)	86 (17.2)	26 (5.3)	69 (13.8)
None of the time	50 (10.0)	32 (6.5)	68 (13.7)	35 (8.7)	54 (14.5)	25 (7.5)	30 (6.0)	25 (5.0)	58 (11.7)
Census division, n (%)									
New England	14 (2.9)	21 (4.3)	11 (2.1)	11 (2.7)	8 (2.2)	16 (4.8)	3 (0.5)	22 (4.3)	23 (4.6)
Middle Atlantic	19 (3.8)	73 (14.6)	70 (14.1)	39 (9.8)	61 (16.3)	71 (21.0)	12 (2.4)	72 (14.4)	65 (13.0)
East North Central	40 (8.1)	38 (7.5)	58 (11.6)	29 (7.2)	21 (5.6)	46 (13.8)	21 (4.1)	88 (17.5)	55 (11.0)
West North Central	27 (5.4)	17 (3.3)	27 (5.4)	10 (2.5)	3 (0.8)	17 (5.1)	9 (1.8)	50 (9.9)	19 (3.8)
South Atlantic	61 (12.2)	71 (14.2)	187 (37.4)	55 (13.6)	93 (24.8)	44 (13.0)	26 (5.2)	97 (19.5)	101 (20.3)
East South Central	17 (3.4)	8 (1.7)	31 (6.1)	2 (0.6)	6 (1.5)	3 (1.0)	3 (0.7)	24 (4.8)	15 (3.1)
West South Central	87 (17.4)	37 (7.5)	75 (15.0)	88 (21.8)	75 (20.0)	38 (11.2)	25 (5.0)	49 (9.8)	42 (8.5)
Mountain	106 (21.2)	26 (5.2)	15 (3.1)	59 (14.7)	27 (7.1)	33 (9.9)	93 (18.6)	30 (5.9)	57 (11.4)
Pacific	128 (25.6)	208 (41.6)	26 (5.2)	110 (27.3)	81 (21.6)	68 (20.2)	308 (61.7)	69 (13.7)	122 (24.4)
Residence urbanicity, n (%)									
Big city	88 (17.6)	108 (21.5)	156 (31.2)	114 (28.4)	155 (41.5)	96 (28.7)	98 (19.7)	53 (10.6)	110 (21.9)
Smaller city	72 (14.5)	73 (14.7)	97 (19.4)	86 (21.3)	102 (27.5)	35 (10.3)	88 (17.5)	83 (16.6)	99 (19.7)
Suburban area	135 (27.1)	257 (51.3)	155 (31.1)	129 (32.0)	65 (17.5)	147 (43.7)	142 (28.4)	174 (34.8)	163 (32.6)
Small town	59 (11.8)	32 (6.3)	37 (7.4)	39 (9.8)	36 (9.7)	23 (7.0)	84 (16.7)	98 (19.6)	57 (11.4)
Rural	145 (29.0)	31 (6.2)	55 (11.0)	34 (8.5)	15 (3.9)	35 (10.3)	88 (17.7)	92 (18.4)	72 (14.4)

*AIAN* American Indian or Alaska Native, *MENA* Middle Eastern or North African, *NHPI* Native Hawaiian or Pacific Islander, *IQR* interquartile range, *BMI* body mass index, *GED* general education development

a Chronic conditions include: asthma; autoimmune/immunocompromised; history of cancer (excluding skin cancer); chronic kidney disease or on dialysis; chronic liver disease; chronic lung disease; diabetes (type 1 or type 2); heart conditions; high blood pressure or hypertension; immune deficiency, HIV, or AIDS; obesity; sickle cell disease; solid organ transplant recipient; and stroke

b Calculated from self-reported height (feet and inches) and weight (pounds)

c Disabilities include: deafness or difficulty hearing; blindness or difficulty seeing; difficulty concentrating, remembering, or making decisions; difficulty walking or climbing stairs; difficulty dressing or bathing; and difficulty doing errands alone

d Responded to the question ‘How well do you speak English’ with ‘fairly well’, ‘not at all’, or ‘poorly’

e Unemployed includes individuals who reported their employment status as: temporarily laid off, unemployed, permanently disabled, homemaker, student, and other

**Table 2:** Adherence to preventive care guidelines by race and/or ethnicity, weighted to be nationally representative within racial and/or ethnic populations: United States, June – August 2023 (*N* = 4,111)

	AIAN	Asian	Black	English-speaking Latino	Spanish-speaking Latino	MENA	NHPI	White	Multiracial
Blood pressure screening	428 (85.6)	436 (87.2)	430 (86.0)	332 (82.6)	333 (89.4)	273 (81.2)	418 (83.7)	443 (88.6)	420 (84.0)
Cholesterol screening	370 (77.3)	398 (85.2)	392 (82.5)	314 (81.6)	325 (88.5)	266 (83.0)	388 (81.9)	381 (86.5)	385 (80.4)
Breast cancer screening	94 (67.1)	88 (70.9)	108 (75.2)	68 (70.6)	76 (84.4)	37 (53.4)	87 (72.1)	100 (69.8)	74 (60.9)
Cervical cancer screening	103 (66.9)	128 (66.0)	131 (77.4)	75 (70.6)	119 (76.3)	60 (55.7)	110 (69.7)	88 (66.0)	124 (69.4)
Colorectal cancer screening	183 (69.4)	177 (74.6)	205 (76.4)	130 (65.7)	127 (79.0)	102 (64.8)	175 (69.3)	208 (74.5)	144 (64.2)
Influenza vaccine	232 (46.5)	304 (60.8)	273 (54.6)	206 (51.3)	198 (53.0)	139 (41.4)	268 (53.6)	301 (60.1)	229 (45.8)
Pneumococcal vaccine	65 (56.3)	75 (64.5)	86 (75.6)	64 (72.8)	27 (52.9)	56 (68.0)	81 (72.7)	133 (78.0)	63 (69.9)
Shingles vaccine	49 (42.6)	68 (58.7)	49 (43.6)	44 (49.9)	25 (50.1)	43 (53.0)	67 (61.0)	104 (61.0)	27 (29.6)

*AIAN* American Indian or Alaska Native, *MENA* Middle Eastern or North African, *NHPI* Native Hawaiian or Pacific Islander

**Table 3:** Adjusted prevalence ratios (and 95% confidence interval)^a^ between race and/or ethnicity and adherence to preventive care, weighted to be nationally representative within racial and/or ethnic populations: United States, June – August 2023 (*N* = 4,111)

	Latino								
	AIAN	Asian	Black	English-speaking	Spanish-speaking	MENA	NHPI	White	Multiracial
Blood pressure screening	0.90 (0.78, 1.04)	1.05 (0.91, 1.20)	0.97 (0.84, 1.12)	0.98 (0.84, 1.13)	1.07 (0.92, 1.25)	0.99 (0.85, 1.16)	0.97 (0.84, 1.12)	1.0 (ref)	0.96 (0.83, 1.11)
Cholesterol screening	0.98 (0.85, 1.12)	1.04 (0.91, 1.18)	0.99 (0.86, 1.13)	0.96 (0.84, 1.11)	1.05 (0.91, 1.21)	0.94 (0.81, 1.10)	0.96 (0.84, 1.10)	1.0 (ref)	0.97 (0.85, 1.11)
Breast cancer screening	1.00 (0.75, 1.32)	1.08 (0.81, 1.44)	1.10 (0.84, 1.45)	1.05 (0.77, 1.43)	1.26 (0.93, 1.70)	0.79 (0.54, 1.16)	1.07 (0.80, 1.43)	1.0 (ref)	0.92 (0.68, 1.24)
Cervical cancer screening	1.02 (0.76, 1.35)	1.02 (0.77, 1.34)	1.17 (0.89, 1.54)	1.08 (0.80, 1.48)	1.16 (0.88, 1.52)	0.85 (0.61, 1.18)	1.06 (0.80, 1.40)	1.0 (ref)	1.05 (0.80, 1.38)
Colorectal cancer screening	0.96 (0.79, 1.18)	1.07 (0.88, 1.31)	1.07 (0.88, 1.30)	0.93 (0.75, 1.16)	1.13 (0.90, 1.41)	0.91 (0.71, 1.15)	0.97 (0.79, 1.19)	1.0 (ref)	0.91 (0.73, 1.12)
Influenza vaccine	0.82 (0.69, 0.98)	1.15 (0.98, 1.35)	0.97 (0.83, 1.15)	0.94 (0.79, 1.12)	1.03 (0.86, 1.24)	0.75 (0.62, 0.92)	0.96 (0.81, 1.13)	1.0 (ref)	0.84 (0.71, 1.00)
Pneumococcal vaccine	0.80 (0.59, 1.08)	0.91 (0.69, 1.22)	1.02 (0.77, 1.33)	1.03 (0.76, 1.39)	0.78 (0.51, 1.19)	0.97 (0.71, 1.34)	0.95 (0.72, 1.26)	1.0 (ref)	0.97 (0.72, 1.31)
Shingles vaccine	0.76 (0.54, 1.08)	1.04 0.77, 1.42)	0.75 (0.54, 1.06)	0.89 (0.62, 1.26)	0.92 (0.59, 1.42)	0.93 (0.65, 1.33)	1.03 (0.76, 1.40)	1.0 (ref)	0.53 (0.34, 0.81)

*AIAN* American Indian or Alaska Native, *MENA* Middle Eastern or North African, *NHPI* Native Hawaiian or Pacific Islander

a Adjusted for age, sex (excluding breast and cervical cancer screenings), chronic health conditions, and self-reported health

**Table 4:** Difference between adherence to cancer screenings and influenza vaccination guidelines in PREV-CARE survey participants and Healthy People 2030 targets^a^, stratified by race and/or ethnicity

	*N*	Breast cancer screening	Cervical cancer screening	Colorectal cancer screening	Influenza vaccine
AIAN	500	−13.2	−12.3	−3.4	−23.5
Asian	500	−9.4	−13.2	1.8	−9.2
Black	500	−4.8	−1.8	3.6	−15.4
Latino, English-speaking	402	−9.7	−8.6	−7.1	−18.7
Latino, Spanish-speaking	373	4.1	−2.9	6.2	−17.0
MENA	336	−26.9	−23.5	−8.0	−28.6
NHPI	500	−8.2	−9.5	−3.5	−16.4
White	500	−10.5	−13.2	1.7	−9.9
Multiracial	500	−19.4	−9.8	−8.6	−24.2

Adherence estimates were weighted to be nationally representative within racial and/or ethnic populations

*AIAN* American Indian or Alaska Native, *MENA* Middle Eastern or North African, *NHPI* Native Hawaiian or Pacific Islander

a *Healthy People 2030 targets*: breast cancer screening (80.3%), cervical cancer screening (79.2%), colorectal cancer screening (72.8%), and influenza vaccination (70.0%)[5]

## Data Availability

Data and code used to conduct this analysis are available upon reasonable request. Please contact the corresponding author (P Strassle).
