# Supplementary material for: Racial and/or Ethnic Differences in Adherence to Preventive Care Guidelines among US Adults Using a Nationally Representative Survey of All Eight Major Racial and/or Ethnic Populations
Source: J Racial Ethn Health Disparities. Author manuscript; Available in PMC 2026 Oct 3. (PMC13633694; doi:10.1007/s40615-026-03159-9)
Supplement: Supplementary file 1 [file NIHMS2210613-supplement-Supplement.docx]

**Supplemental Materials**

**Supplemental Table 1**. Preventive services guidelines recommended by the U.S. Preventive Services Task Force (USPSTF) and the CDC Advisory Committee on Immunization Practices (ACIP) in 2023.

|  | **Population^a^** | **Frequency** |
| --- | --- | --- |
| **Vaccinations** |  |  |
| Influenza | All adults | Yearly |
| Pneumococcal | All adults 65+ | Once after 65 |
| Shingles | All adults 65+ | Once after 65 |
| **Cancer Screening** |  |  |
| Breast | Women 50-74 | 2 years |
| Cervical^c^ | Women 35-64 | 5 years |
| Colorectal | All adults 50-74 | Varies by test^d^ |
| **Other Screenings** |  |  |
| Blood pressure | All adults 35+ | 2 years |
| Cholesterol | All adults 35-74 | 5 years |
| ^a^ Unless contraindicated or inappropriate (e.g., previous hysterectomy for cervical cancer screening)  ^c^ Pap or HPV testing in the past 5 years  ^d^ Every 1, 5, or 10 years based on screening test (fecal occult blood test: 1 year; flexible sigmoidoscopy: 5 years; colonoscopy: 10 years) | | |

Note: The preventive guidelines included above were in use during the study period (2023). In 2024, ACIP updated their recommendations for pneumococcal vaccination, lowering the age from 65 to 50 years old.^1^ The USPSTF also updated their recommendations in 2024, lowering the age for breast cancer screening from 50 to 40 years old^2^ and for colorectal cancer screening from 50 to 45 years old.^3^

**Supplemental Table 2.** Number and proportion of individuals eligible for each screening guideline (unweighted), stratified by race and/or ethnicity.

|  | **Blood Pressure Screening** | **Cholesterol Screening** | **Breast Cancer Screening** | **Cervical**  **Cancer**  **Screening** | **Colorectal**  **Cancer**  **Screening** | **Influenza**  **Vaccine** | **Pneumo-**  **coccal**  **Vaccine** | **Shingles**  **Vaccine** |
| --- | --- | --- | --- | --- | --- | --- | --- | --- |
| **Total eligible adults, N** | **4,111** | **3,886** | **1,358** | **1,049** | **2,040** | **4,111** | **936** | **936** |
| **Race and/or ethnicity** |  |  |  |  |  |  |  |  |
| AIAN | 500 (100.0) | 475 (95.0) | 140 (28.2) | 167 (36.6) | 261 (53.4) | 500 (100.0) | 110 (22.0) | 110 (22.0) |
| Asian | 500 (100.0) | 470 (94.0) | 133 (26.9) | 198 (40.7) | 241 (48.4) | 500 (100.0) | 116 (23.2) | 116 (23.2) |
| Asian Indian | 80 (100.0) | 76 (95.0) | 15 (18.8) | 28 (35.0) | 31 (38.8) | 80 (100.0) | 18 (22.5) | 18 (22.5) |
| Chinese | 156 (100.0) | 144 (92.3) | 42 (26.9) | 50 (32.1) | 81 (51.9) | 156 (100.0) | 47 (30.1) | 47 (30.1) |
| Filipino | 58 (100.0) | 55 (94.8) | 19 (32.8) | 29 (50.0) | 27 (46.6) | 58 (100.0) | 7 (12.1) | 7 (12.1) |
| Japanese | 94 (100.0) | 86 (91.5) | 38 (40.4) | 38 (40.4) | 64 (68.1) | 94 (100.0) | 35 (37.2) | 35 (37.2) |
| Korean | 44 (100.0) | 42 (95.5) | 9 (20.5) | 22 (50.0) | 15 (34.1) | 44 (100.0) | 5 (11.4) | 5 (11.4) |
| Other Asian | 68 (100.0) | 67 (98.5) | 10 (14.7) | 31 (45.6) | 23 (33.8) | 68 (100.0) | 4 (5.9) | 4 (5.9) |
| Black | 500 (100.0) | 469 (93.8) | 164 (33.3) | 166 (36.6) | 291 (59.8) | 500 (100.0) | 142 (28.4) | 142 (28.4) |
| Latino |  |  |  |  |  |  |  |  |
| English-speaking | 492 (100.0) | 479 (97.4) | 132 (27.3) | 170 (37.5) | 251 (52.2) | 492 (100.0) | 101 (20.5) | 101 (20.5) |
| Spanish-speaking | 283 (100.0) | 278 (98.2) | 78 (27.8) | 143 (57.2) | 125 (44.3) | 283 (100.0) | 33 (11.7) | 33 (11.7) |
| Cuban | 49 (100.0) | 45 (91.8) | 15 (30.6) | 17 (34.7) | 28 (57.1) | 49 (100.0) | 16 (32.7) | 16 (32.7) |
| Mexican | 386 (100.0) | 380 (98.4) | 91 (23.6) | 169 (43.8) | 168 (43.5) | 386 (100.0) | 60 (15.5) | 60 (15.5) |
| Puerto Rican | 138 (100.0) | 133 (96.4) | 56 (40.6) | 40 (29.0) | 86 (62.3) | 138 (100.0) | 33 (23.9) | 33 (23.9) |
| South American | 91 (100.0) | 89 (97.8) | 23 (25.3) | 35 (38.5) | 45 (49.5) | 91 (100.0) | 11 (12.1) | 11 (12.1) |
| Other Latino | 111 (100.0) | 110 (99.1) | 25 (22.5) | 52 (46.8) | 49 (44.1) | 111 (100.0) | 14 (12.6) | 14 (12.6) |
| MENA | 366 (100.0) | 325 (96.7) | 73 (22.0) | 148 (46.4) | 142 (42.9) | 366 (100.0) | 64 (19.1) | 64 (19.1) |
| NHPI | 500 (100.0) | 481 (96.2) | 141 (28.8) | 197 (43.6) | 251 (50.7) | 500 (100.0) | 89 (17.8) | 89 (17.8) |
| Guamanian or Chamorro | 51 (100.0) | 49 (96.1) | 22 (43.1) | 27 (52.9) | 26 (51.0) | 51 (100.0) | 8 (15.7) | 8 (15.7) |
| Native Hawaiian | 247 (100.0) | 236 (95.5) | 77 (31.2) | 82 (33.2) | 138 (55.9) | 247 (100.0) | 52 (21.1) | 52 (21.1) |
| Samoan | 60 (100.0) | 59 (98.3) | 13 (21.7) | 27 (45.0) | 26 (43.3) | 60 (100.0) | 3 (5.0) | 3 (5.0) |
| Other Pacific Islander | 142 (100.0) | 137 (96.5) | 29 (20.4) | 61 (43.0) | 61 (43.0) | 142 (100.0) | 26 (18.3) | 26 (18.3) |
| White | 500 (100.0) | 445 (89.0) | 163 (33.3) | 134 (29.1) | 311 (62.8) | 500 (100.0) | 169 (33.8) | 169 (33.8) |
| Multiracial | 500 (100.0) | 475 (95.0) | 121 (24.4) | 186 (40.3) | 226 (45.6) | 500 (100.0) | 91 (18.2) | 91 (18.2) |
| Abbreviations: AIAN, American Indian or Alaska Native; MENA, Middle Eastern or North African; NHPI, Native Hawaiian or Pacific Islander  ^a^ 55 females reported having both breasts removed or a history of breast cancer and were ineligible for breast cancer screening; AIAN: 4; Asian: 5; Black: 7; English-speaking Latino: 9; Spanish-speaking Latino: 2; MENA: 4; NHPI: 10; White: 11; Multiracial: 3.  ^b^ 319 females reported having a hysterectomy or a history of cervical cancer and were ineligible for cervical cancer screening; AIAN: 44; Asian: 14; Black: 47; English-speaking Latino: 39; Spanish-speaking Latino: 33; MENA: 17; NHPI: 48; White: 39; Multiracial: 38.  ^c^ 57 individuals reported having their entire colon removed or a history of colon cancer and were ineligible for colorectal cancer screening; AIAN: 11; Asian: 2; Black: 13; English-speaking Latino: 11; Spanish-speaking Latino: 1; MENA: 5; NHPI: 5; White: 5; Multiracial: 4. | | | | | | | | |

**Supplemental Table 3.** Adherence to preventive care guidelines among Asian adults, stratified by ethnicity and/or country of origin, weighted to be nationally representative within racial and/or ethnic populations: United States, June – August 2023 (N=500).

|  | **Overall**  **(n=500)** | **Asian Indian**  **(n=80)** | **Chinese**  **(n=156)** | **Filipino**  **(n=58)** | **Japanese**  **(n=94)** | **Korean**  **(n=44)** | **Other Asian**  **(n=68)** |
| --- | --- | --- | --- | --- | --- | --- | --- |
| **Blood pressure screening** | 436 (87.2) | 74 (89.0) | 128 (85.6) | 52 (92.7) | 82 (85.6) | 42 (91.0) | 60 (83.0) |
| **Cholesterol screening** | 398 (85.2) | 65 (80.8) | 113 (85.3) | 46 (86.3) | 75 (86.8) | 37 (84.4) | 62 (87.7) |
| **Breast cancer screening** | 88 (70.9) | 18 (84.8) | 28 (84.0) | 11 (90.6) | 21 (53.2) | N/A | 6 (70.0) |
| **Cervical cancer screening** | 128 (66.0) | 19 (61.8) | 26 (54.4) | 22 (78.6) | 26 (71.7) | 13 (55.1) | 23 (79.7) |
| **Colorectal cancer screening** | 177 (74.6) | 29 (81.0) | 53 (73.5) | 23 (91.9) | 44 (72.4) | 12 (71.4) | 15 (59.4) |
| **Influenza vaccine** | 304 (60.8) | 44 (53.5) | 98 (65.6) | 42 (74.7) | 55 (57.8) | 23 (51.6) | 41 (57.4) |
| **Pneumococcal vaccine** | 75 (64.5) | 13 (82.7) | 30 (61.8) | N/A | 21 (62.3) | N/A | N/A |
| **Shingles vaccine** | 68 (58.7) | 6 (38.5) | 36 (73.7) | N/A | 15 (43.7) | N/A | N/A |

Abbreviations: N/A, not analyzed due to small sample size (eligible population <10 adults [unweighted])

**Supplemental Table 4.** Adherence to preventive care guidelines among Latino adults, stratified by ethnicity and/or country of origin, weighted to be nationally representative within racial and/or ethnic populations: United States, June – August 2023 (N=500).

|  | **Overall**  **(n=775)** | **Cuban**  **(n=49)** | **Mexican^a^**  **(n=386)** | **Puerto Rican**  **(n=138)** | **South American**  **(n=91)** | **Other Latino**  **(n=111)** |
| --- | --- | --- | --- | --- | --- | --- |
| **Blood pressure screening** | 665 (85.8) | 38 (87.1) | 319 (83.4) | 122 (89.4) | 91 (90.5) | 94 (85.2) |
| **Cholesterol screening** | 639 (84.9) | 35 (91.3) | 302 (80.2) | 121 (93.2) | 86 (86.8) | 95 (87.5) |
| **Breast cancer screening** | 144 (77.3) | 7 (73.8) | 58 (71.2) | 47 (88.9) | 17 (75.4) | 15 (75.1) |
| **Cervical cancer screening** | 194 (74.0) | 9 (75.4) | 95 (66.3) | 28 (88.2) | 24 (80.4) | 37 (83.8) |
| **Colorectal cancer screening** | 257 (71.7) | 19 (85.7) | 101 (62.4) | 76 (90.7) | 31 (69.9) | 30 (64.8) |
| **Influenza vaccine** | 404 (52.1) | 16 (37.5) | 200 (52.1) | 81 (59.3) | 50 (49.6) | 57 (51.3) |
| **Pneumococcal vaccine** | 90 (65.5) | 6 (39.2) | 42 (75.5) | 25 (59.9) | 7 (56.6) | 10 (80.2) |
| **Shingles vaccine** | 69 (50.0) | 9 (58.6) | 28 (50.6) | 23 (56.5) | 6 (43.8) | 3 (22.5) |

^a^ Includes individuals who identified as Mexican, Mexican American, or Chicano

**Supplemental Table 5.** Adherence to preventive care guidelines among Pacific Islander adults, stratified by ethnicity and/or country of origin, weighted to be nationally representative within racial and/or ethnic populations: United States, June – August 2023 (N=500).

|  | **Overall**  **(n=500)** | **Guamanian or Chamorro**  **(n=51)** | **Native Hawaiian**  **(n=247)** | **Samoan**  **(n=60)** | **Other Pacific Islander**  **(n=142)** |
| --- | --- | --- | --- | --- | --- |
| **Blood pressure screening** | 418 (83.7) | 48 (97.8) | 213 (83.6) | 49 (79.7) | 108 (80.4) |
| **Cholesterol screening** | 388 (81.9) | 41 (91.5) | 190 (79.7) | 51 (83.2) | 105 (81.9) |
| **Breast cancer screening** | 87 (72.1) | 18 (81.2) | 42 (64.1) | 6 (71.1) | 21 (86.0) |
| **Cervical cancer screening** | 110 (69.7) | 18 (76.8) | 50 (73.6) | 12 (54.9) | 31 (67.2) |
| **Colorectal cancer screening** | 175 (69.3) | 18 (67.6) | 94 (69.3) | 19 (64.8) | 44 (72.1) |
| **Influenza vaccine** | 268 (53.6) | 29 (58.4) | 130 (51.1) | 33 (53.1) | 76 (57.0) |
| **Pneumococcal vaccine** | 81 (72.7) | N/A | 40 (63.8) | N/A | 23 (78.1) |
| **Shingles vaccine** | 67 (61.0) | N/A | 38 (59.4) | N/A | 14 (45.9) |

Abbreviations: N/A, not analyzed due to small sample size (eligible population <10 adults [unweighted])

**Supplemental Table 6.** Adherence to preventive care guidelines, stratified by sociodemographics, weighted to be nationally representative within racial and/or ethnic populations.

|  | **Blood Pressure Screening** | **Cholesterol Screening** | **Breast Cancer Screening** | **Cervical**  **Cancer**  **Screening** | **Colorectal**  **Cancer**  **Screening** | **Influenza**  **Vaccine** | **Pneumo-**  **coccal**  **Vaccine** | **Shingles**  **Vaccine** |
| --- | --- | --- | --- | --- | --- | --- | --- | --- |
| **Eligible adults, N** | **4,111** | **3,886** | **1,358** | **1,049** | **2,040** | **4,111** | **936** | **936** |
| **Age group** |  |  |  |  |  |  |  |  |
| 35-39 | 465 (78.1) | 433 (72.8) | N/A | 196 (69.0) | N/A | 251 (42.2) | N/A | N/A |
| 40-44 | 536 (80.3) | 509 (76.1) | N/A | 222 (71.6) | N/A | 310 (46.4) | N/A | N/A |
| 45-49 | 433 (83.6) | 418 (80.7) | N/A | 161 (69.1) | N/A | 216 (41.7) | N/A | N/A |
| 50-54 | 436 (85.2) | 426 (83.2) | 147 (60.3) | 121 (61.9) | 271 (53.6) | 239 (46.7) | N/A | N/A |
| 55-59 | 373 (87.6) | 366 (86.0) | 140 (69.0) | 121 (73.3) | 289 (69.8) | 223 (52.3) | N/A | N/A |
| 60-64 | 399 (87.7) | 402 (88.5) | 161 (69.6) | 117 (69.1) | 321 (74.6) | 273 (60.1) | N/A | N/A |
| 65-69 | 397 (91.3) | 397 (91.5) | 158 (71.9) | N/A | 341 (80.3) | 251 (57.7) | 243 (55.9) | 181 (41.7) |
| 70-74 | 257 (93.0) | 266 (96.3) | 125 (83.9) | N/A | 228 (86.0) | 205 (74.2) | 219 (79.3) | 149 (54.0) |
| 75+ | 216 (96.1) | N/A | N/A | N/A | N/A | 182 (80.7) | 186 (82.7) | 147 (65.3) |
| **Sex** |  |  |  |  |  |  |  |  |
| Female | 1,117 (52.2) | 1,673 (82.9) | 732 (69.8) | 939 (69.1) | 775 (72.1) | 1,117 (52.2) | 369 (72.2) | 265 (51.9) |
| Male | 1,643 (83.4) | 1,546 (82.8) | N/A | N/A | 675 (69.9) | 1,033 (52.4) | 279 (65.7) | 212 (49.9) |
| **Self-reported general health** |  |  |  |  |  |  |  |  |
| Excellent\very good\good | 2,145 (85.8) | 1,982 (83.7) | 453 (68.0) | 550 (68.3) | 928 (70.6) | 1,355 (54.2) | 421 (71.3) | 285 (48.2) |
| Poor\Fair | 1,368 (85.0) | 1,236 (81.4) | 279 (72.9) | 389 (70.3) | 522 (71.9) | 795 (49.4) | 227 (65.7) | 192 (55.7) |
| **Chronic health conditions^a^** |  |  |  |  |  |  |  |  |
| Any chronic condition | 2,294 (91.7) | 2,062 (89.0) | 526 (74.1) | 502 (72.0) | 1,084 (76.0) | 1,473 (58.9) | 550 (75.4) | 396 (54.3) |
| No reported conditions | 1,220 (75.8) | 1,156 (73.6) | 206 (60.8) | 437 (66.1) | 367 (59.7) | 677 (42.1) | 98 (47.4) | 81 (39.4) |
| **Disability^b^** |  |  |  |  |  |  |  |  |
| Any disability | 1,162 (90.1) | 1,024 (86.6) | 272 (70.8) | 224 (65.9) | 554 (74.3) | 704 (54.6) | 278 (72.6) | 177 (46.3) |
| No reported disability | 2,352 (83.3) | 2,194 (81.2) | 460 (69.2) | 715 (70.2) | 896 (69.3) | 1,446 (51.2) | 371 (66.9) | 300 (54.2) |
| **Health insurance** |  |  |  |  |  |  |  |  |
| Any private | 1,695 (88.6) | 1,578 (86.0) | 338 (74.4) | 543 (76.9) | 645 (71.7) | 1,066 (55.7) | 188 (68.6) | 175 (63.5) |
| Public insurance only | 1,524 (88.4) | 1,347 (85.0) | 369 (71.5) | 311 (67.0) | 736 (76.4) | 945 (54.8) | 434 (70.1) | 289 (46.6) |
| Uninsured | 284 (61.7) | 282 (62.3) | 22 (29.2) | 84 (44.9) | 65 (37.6) | 130 (28.4) | 24 (61.4) | 13 (32.3) |
| **English proficiency^c^** |  |  |  |  |  |  |  |  |
| Limited | 240 (80.8) | 229 (80.2) | 41 (76.1) | 75 (68.4) | 87 (70.9) | 131 (44.1) | 31 (58.7) | 27 (51.7) |
| Not limited | 3,273 (85.8) | 2,989 (83) | 691 (69.4) | 864 (69.2) | 1,364 (71.1) | 2,019 (52.9) | 617 (69.9) | 450 (50.9) |
| **Education** |  |  |  |  |  |  |  |  |
| Less than high school graduate | 223 (85.1) | 204 (82.8) | 50 (76.8) | 54 (68) | 82 (66.5) | 134 (51.0) | 42 (63.1) | 24 (36.6) |
| High school diploma/GED | 1,071 (80.8) | 1,014 (80.9) | 240 (68.1) | 229 (57.3) | 457 (66.7) | 647 (48.8) | 211 (64.4) | 159 (48.7) |
| Some college/vocational school | 995 (86.7) | 892 (81.9) | 213 (65.9) | 275 (70.2) | 417 (71.3) | 543 (47.3) | 173 (70.1) | 114 (46.2) |
| College graduate | 731 (87.9) | 657 (82.7) | 139 (72.0) | 230 (75.0) | 295 (74.3) | 472 (56.7) | 126 (73.1) | 97 (56.3) |
| Graduate or advanced degree | 494 (90.8) | 452 (89.6) | 90 (78.1) | 152 (83.6) | 200 (79.8) | 355 (65.2) | 96 (78.3) | 82 (66.9) |
| **Family annual income** |  |  |  |  |  |  |  |  |
| <$20,000 | 579 (79.8) | 565 (77.9) | 145 (20.1) | 156 (23.8) | 235 (32.9) | 323 (44.5) | 86 (11.8) | 59 (8.2) |
| $20,000-$59,999 | 1288 (85.4) | 1150 (76.3) | 282 (19.0) | 320 (22.7) | 512 (34.4) | 760 (50.4) | 258 (17.1) | 164 (10.9) |
| $60,000-$99,999 | 713 (86.2) | 671 (81.1) | 130 (15.9) | 200 (25.4) | 299 (27.2) | 465 (56.3) | 126 (15.2) | 96 (11.6) |
| ≥$100,000 | 656 (91.8) | 587 (82.2) | 107 (15.1) | 176 (26.6) | 290 (40.9) | 427 (59.8) | 109 (15.2) | 104 (14.6) |
| **Census division** |  |  |  |  |  |  |  |  |
| New England | 114 (88.4) | 96 (86.0) | 28 (92.6) | 26 (85.9) | 50 (74.7) | 77 (59.8) | 36 (89.4) | 29 (72.5) |
| Middle Atlantic | 425 (88.1) | 405 (88.8) | 84 (69.1) | 104 (65.9) | 168 (71.5) | 276 (57.2) | 65 (63.8) | 49 (48.4) |
| East North Central | 343 (86.9) | 311 (82.7) | 71 (72.1) | 90 (73.5) | 145 (75.0) | 206 (52.0) | 64 (72.3) | 52 (58.6) |
| West North Central | 155 (86.8) | 134 (80.1) | 28 (72.4) | 44 (71.3) | 45 (60.6) | 105 (59.0) | 26 (74.1) | 20 (55.8) |
| South Atlantic | 634 (86.2) | 592 (84.8) | 155 (74.2) | 164 (68.1) | 285 (74.1) | 357 (48.6) | 134 (68.3) | 83 (42.1) |
| East South Central | 97 (87.8) | 88 (82.0) | 19 (66.2) | 24 (54.6) | 41 (73.2) | 57 (51.7) | 9 (51.7) | 8 (44.1) |
| West South Central | 432 (83.9) | 395 (80.6) | 74 (65.5) | 110 (60.9) | 162 (67.2) | 270 (52.3) | 65 (71.2) | 53 (58.3) |
| Mountain | 372 (83.4) | 322 (75.7) | 72 (60.7) | 107 (74.0) | 142 (64.1) | 200 (44.8) | 65 (62.5) | 38 (37.0) |
| Pacific | 942 (84.1) | 877 (83.0) | 201 (69.1) | 271 (71.8) | 412 (72.7) | 603 (53.8) | 184 (70.5) | 145 (55.6) |
| **Residence urbanicity** |  |  |  |  |  |  |  |  |
| Rural | 879 (85.3) | 794 (82.2) | 195 (66.0) | 213 (66.8) | 388 (71.4) | 518 (50.2) | 187 (67.1) | 125 (44.8) |
| Non-rural | 2,634 (85.5) | 2,424 (83.0) | 538 (71.3) | 726 (69.8) | 1,062 (71.0) | 1,632 (53.0) | 461 (70.1) | 352 (53.6) |
| Abbreviations: AIAN, American Indian or Alaska Native; MENA, Middle Eastern or North African; NHPI, Native Hawaiian or Pacific Islander; GED, general education development  ^a^ Chronic conditions include: asthma; autoimmune/immunocompromised; history of cancer (excluding skin cancer); chronic kidney disease or on dialysis; chronic liver disease; chronic lung disease; diabetes (type 1 or type 2); heart conditions; high blood pressure or hypertension; immune deficiency, HIV, or AIDS; obesity; sickle cell disease; solid organ transplant recipient; and stroke  ^b^ Disabilities include: deafness or difficulty hearing; blindness or difficulty seeing; difficulty concentrating, remembering, or making decisions; difficulty walking or climbing stairs; difficulty dressing or bathing; and difficulty doing errands alone  ^c^ All participants were asked ‘How well do you speak English?’ Individuals who responded with ‘not at all’ or ‘poorly’ were classified as low English proficiency | | | | | | | | |

**Supplemental Table 7.** Difference between adherence to cancer screenings and influenza vaccination guidelines among Asian, Latino, and NHPI participants and Healthy People 2030 targets^a^, stratified by ethnicity/national origin. Adherence estimates were weighted to be nationally representative within racial and/or ethnic populations.

|  | **N** | **Breast**  **Cancer**  **Screening** | **Cervical**  **Cancer**  **Screening** | **Colorectal**  **Cancer**  **Screening** | **Influenza**  **Vaccine** |
| --- | --- | --- | --- | --- | --- |
| **Asian** | 500 | -9.4 | -13.2 | 1.8 | -9.2 |
| Asian Indian | 83 | 4.5 | -17.4 | 8.2 | -16.5 |
| Chinese | 149 | 3.7 | -24.8 | 0.7 | -4.4 |
| Filipino | 56 | 10.3 | -0.6 | 19.1 | 5.7 |
| Japanese | 95 | -27.1 | -7.5 | -0.4 | -12.2 |
| Korean | 46 | N/A | -24.1 | -1.4 | -18.4 |
| Other Asian | 72 | -10.3 | 0.5 | -13.4 | -12.6 |
| **Latino** | 775 |  |  |  |  |
| Cuban/Cuban American | 49 | -6.5 | -3.8 | 12.9 | -32.5 |
| Mexican^b^ | 386 | -9.1 | -12.9 | -10.4 | -17.9 |
| Puerto Rican | 138 | 8.6 | 9.0 | 17.9 | -10.7 |
| South American | 91 | -4.9 | 1.2 | -2.9 | -20.4 |
| Other Latino | 111 | -5.2 | 4.6 | -8.0 | -18.7 |
| **Native Hawaiian or Pacific Islander** | 500 | -8.2 | -9.5 | -3.5 | -16.4 |
| Guamanian or Chamorro | 51 | 0.9 | -2.4 | -5.2 | -11.6 |
| Native Hawaiian | 247 | -16.2 | -5.6 | -3.5 | -18.9 |
| Samoan | 60 | -9.2 | -24.3 | -8.0 | -16.9 |
| Other Pacific Islander | 142 | 5.7 | -12.0 | -0.7 | -13.0 |
| Abbreviations: N/A, not analyzed due to small sample size (eligible population <10 adults [unweighted])  ^a^ *Healthy People 2030 targets*: breast cancer screening (80.3%), cervical cancer screening (79.2%), colorectal cancer screening (72.8%), and influenza vaccination (70.0%)[5]  ^b^ Includes individuals who identified as Mexican, Mexican American, or Chicano | | | | | |

**Supplemental Table 8**. Prevalence of adherence to all recommended preventive care guidelines, overall and stratified by race and/or ethnicity, and adjusted association between race and/or ethnicity and adherence to all recommended guidelines.

|  | **Adherence to All Recommended Guidelines** | |
| --- | --- | --- |
|  | **N (%)** | **PR (95% CI)** |
| **Overall** | 1,414 (34.4) | – |
| **American Indian or Alaska Native** | 142 (28.3) | 0.69 (0.56, 0.86) |
| **Asian** | 191 (38.3) | 1.02 (0.83, 1.24) |
| Asian Indian | 34 (33.4) | 0.81 (0.51, 1.28) |
| Chinese | 65 (41.8) | 1.0 (ref) |
| Filipino | 27 (55.7) | 1.39 (0.89, 2.16) |
| Japanese | 36 (31.3) | 0.75 (0.48, 1.18) |
| Korean | 17 (30.3) | 0.75 (0.42, 1.38) |
| Other Asian | 28 (37.2) | 0.92 (0.58, 1.47) |
| **Black** | 179 (35.8) | 0.90 (0.73, 1.10) |
| **Latino** |  |  |
| English-speaking | 134 (33.3) | 0.84 (0.67, 1.04) |
| Spanish-speaking | 142 (38.1) | 1.00 (0.81, 1.24) |
| Cuban | 14 (18.5) | 0.55 (0.27, 1.12) |
| Mexican^b^ | 130 (34.4) | 1.0 (ref) |
| Puerto Rican | 59 (44.0) | 1.26 (0.92, 1.72) |
| South American | 31 (35.7) | 1.05 (0.73, 1.53) |
| Other Latino | 38 (36.4) | 1.07 (0.73, 1.53) |
| **Middle Eastern or North African** | 86 (25.7) | 0.64 (0.50, 0.82) |
| **Native Hawaiian or Pacific Islander** | 170 (34.1) | 0.84 (0.69, 1.03) |
| Guamanian or Chamorro | 19 (43.5) | 1.29 (0.80, 2.08) |
| Native Hawaiian | 87 (35.5) | 1.0 (ref) |
| Samoan | 14 (26.9) | 0.73 (0.43, 1.25) |
| Other Pacific Islander | 41 (31.2) | 0.95 (0.65, 1.38) |
| **White** | 212 (42.3) | 1.0 (ref) |
| **Multiracial** | 157 (31.5) | 0.79 (0.64, 0.97) |
| Abbreviations: PR, prevalence ratio; CI, confidence interval  ^a^ Adjusted for age, sex (excluding breast and cervical cancer screenings), chronic health conditions, and self-reported health  ^b^ Includes individuals who identified as Mexican, Mexican American, or Chicano | | |

**References**

1.	Kobayashi M, Leidner AJ, Gierke R, et al. Expanded Recommendations for Use of Pneumococcal Conjugate Vaccines Among Adults Aged ≥50 Years: Recommendations of the Advisory Committee on Immunization Practices — United States, 2024. *Morb Mortal Wkly Rep*. 2025;74:1–8. doi:10.15585/mmwr.mm7401a12.

2. US Preventive Services Task Force. Screening for Breast Cancer: US Preventive Services Task Force Recommendation Statement. JAMA. 2024;331(22):1918–1930. doi:10.1001/jama.2024.5534

3.	US Preventive Services Task Force. Screening for Colorectal Cancer: US Preventive Services Task Force Recommendation Statement. JAMA. 2021;325(19):1965–1977. doi:10.1001/jama.2021.6238
